# Refusals to perform ritual circumcision: a qualitative study of doctors’ professional and ethical reasoning

**DOI:** 10.1186/s12910-020-0444-0

**Published:** 2020-01-10

**Authors:** Liv Astrid Litleskare, Mette Tolås Strander, Reidun Førde, Morten Magelssen

**Affiliations:** Centre for Medical Ethics, Institute of Health and Society, University of Oslo, Oslo, Norway

**Keywords:** Conscientious objection, Professional ethics, Ritual circumcision

## Abstract

**Background:**

Ritual circumcision of infant boys is controversial in Norway, as in many other countries. The procedure became a part of Norwegian public health services in 2015. A new law opened for conscientious objection to the procedure. We have studied physicians’ refusals to perform ritual circumcision as an issue of professional ethics.

**Method:**

Qualitative interview study with 10 urologists who refused to perform ritual circumcision from six Norwegian public hospitals. Interviews were recorded and transcribed, then analysed with systematic text condensation, a qualitative analysis framework.

**Results:**

The physicians are unanimous in grounding their opposition to the procedure in professional standards and norms, based on fundamental tenets of professional ethics. While there is homogeneity in the group when it comes to this reasoning, there are significant variations as to how deeply the matter touches the urologists on a personal level. About half of them connect their stance to their personal integrity, and state that performing the procedure would go against their conscience and lead to pangs of conscience.

**Conclusions:**

It is argued that professional moral norms sometimes might become more or less ‘integrated’ in the professional’s core moral values and moral identity. If this is the case, then the distinction between conscience-based and professional refusals to certain healthcare services cannot be drawn as sharply as it has been.

## Background

After much debate – in the health services, among politicians and in the public square – ritual circumcision of infant boys became a part of Norwegian public health services with a new law which came into effect in 2015 [1]. This meant that circumcision would be performed by urologists and paediatric surgeons in publicly funded hospitals. However, due to not fulfilling national priority setting criteria, there would be a €400 co-payment for the procedure. Prior to the new law, several authorities, including the Medical Ethics Council of the Norwegian Medical Association and the Ombudsman for Children, had warned against the practice [2]. There was also opposition among doctors, with the Norwegian urological association speaking out against the law [3].

When health professionals refuse to partake in healthcare practices to which they have a moral or religious objection, we speak of *conscientious objection* (CO) or *conscientious refusals*. Considerable resistance among hospital clinicians led to a CO clause being included in the law, stating that ‘To the extent that it does not impede proper service, healthcare personnel who for reasons of conscience do not wish to perform or assist in such interventions shall be accommodated’ ([1], §4; our translation). However, whether *conscience* is an apt word here and *conscientious objection* an apt designation for refusals to be involved in ritual circumcision can be questioned. As will be discussed below, it can be argued that ritual circumcision first and foremost challenges *professional norms*, and not (individual) conscience.

In Norway it is Muslim and Jewish parents who want their sons be circumcised. Although ritual circumcision is deeply embedded within Judaism and important also in Islam, it is also prevalent in other populations and countries (such as in the USA), where the practice can be grounded not only in religion but also in tradition, culture and perceived health benefits. In the USA, 81% of men aged 14–59 have been circumcised [4]. In the medical field there has been a ‘trans-Atlantic’ chasm on the issue, with US doctors generally accepting of the practice and emphasizing medical benefits, and European colleagues often being critical of it, emphasizing the risk of harm and questioning the medical evidence for any benefits [5–7]. However, recently there appears to have been an increase in critical views in the US too [8].

There are not many published reports about physicians refusing to partake in ritual circumcision. In an article based on experiences from the Marquette Family Residency Program, USA, Michelle Storms described how ritual circumcision had led to controversy both in the department and among colleagues [9]. Many doctors had felt compelled to identify as ‘conscientious objectors’ to justify their choice to refrain from performing ritual circumcision. The doctors reported ethical and religious reasons for refusing the procedure which the author then likened to reasons being given by doctors who refuse to perform abortion, abortion counselling or vasectomies.

The introduction of the Norwegian law and the conscience clause, together with the strong opposition to the practice voiced by many Norwegian doctors, provided an opportunity to study a topical problem in professional ethics. The aim of the study was twofold: 1) To study a problem in professional medical ethics and physicians’ professional-ethical reasoning; 2) to provide a ‘case study’ of a potentially atypical case of conscientious objection, i.e., refusals to perform ritual circumcision.

## Methods

### Recruitment and characteristics of informants

Information about the study, asking for physicians who refused to perform ritual circumcision to participate as informants, was emailed to department heads at surgical departments in several Norwegian hospitals, then followed up by phone calls. Department heads provided contact information to, in total, three physicians who were willing to be informants; several department heads stated that they were reluctant to disseminate invitations to the study because their physicians were pressed for time. Seven further informants were recruited by informants providing contact with colleagues also willing to participate (‘snowballing’) or through requests via our own professional acquaintances. Ten physicians were recruited in total; we were unable to recruit further informants. All worked at urology departments in six public hospitals in Western, Southern and Eastern Norway; nine were male, one female; nine were consultants and one a urologist in training. Together with urological surgeons, paediatric surgeons also sometimes perform circumcisions, yet we were unable to recruit anyone from the latter group.

### Research interviews

As an important aim of the study was to explore physicians’ professional-ethical reasoning in connection with a complex ethical problem, a qualitative research interview approach was deemed appropriate. From the outset, we intended to interview informants in focus groups, but as we were unable to recruit a sufficient number of informants at each site, semi-structured individual interviews were chosen instead. An interview guide was developed, then adjusted after experiences with the first interviews (see Additional file 1.docx). The interview guide contained questions about informants’ justification for refusing circumcision, about counter-arguments, about potential pressure to partake in circumcision, and about conscientious objection. Interviews lasted 30 to 60 min and were audio recorded and then transcribed. Interviews were performed from February to December 2016.

### Analysis

Transcripts were analyzed by systematic text condensation, a qualitative analysis framework developed by Malterud, building on Giorgi’s method [10]. The method proceeds through four steps. 1) The transcripts were read several times to create an overall impression and note potential themes. Some themes were singled out as particularly promising and relevant. 2) Units of meaning were identified, coded according to topic, and codes were structured in main codes and sub-codes. 3) So-called artificial quotations were constructed from each of the sub-codes. Here, all units of meaning belonging to a sub-code were read together and a ‘quotation’ constructed as representative of the meaning, phrased as if it were an expression made by the informants. 4) The artificial quotations provided the basis for the analytic text, which is supplemented with particularly relevant (genuine) quotations from the interviews. Finally, we returned to the transcripts to ensure that our interpretations were supported by the material.

## Results

All ten informants refused to perform ritual circumcision; three had performed the procedure earlier in their career, but then through experience, reflection and the increased recent attention to the topic had come to change their stance. Due to the way the service was organized, the informants had not necessarily had to register as conscientious objectors; in several of the departments, responsibility for the procedure had either been left to one or two surgeons who were willing to perform it, or it had been outsourced entirely to private hospitals nearby.

### Reasons for refusing and opposing ritual circumcision

Notably, when asked to justify why they refused to perform circumcision, the informants spoke about their arguments against the procedure as such, rather than their own role in it. It therefore seemed that the informants’ primary concern was with what makes circumcision wrong; whereas their own causal contribution to it was only of secondary importance. In other words, they refused to perform circumcision primarily because circumcision to them simply was wrong – not because it would be intolerable for them to contribute to circumcision.

Their reasons for opposing ritual circumcision maps closely to the four traditional principles of healthcare ethics, although none of the informants explicitly used this ethical framework or referred to or named any of the four principles. Concerning *beneficence,* the urologists pointed to the lack of medical indication for ritual circumcision. They were critical towards claims of medically documented benefits from the procedure, and especially that any benefits would be relevant in a Norwegian societal context (e.g., the protective effect against HIV contagion). To differing degrees, the informants were critical of religious and/or cultural justifications for surgical interventions.

With regards to *respect for patient autonomy* it was pointed out that the patient lacked competence, had not requested the procedure, and was also unable to oppose it. *Non-maleficence* encompassed concerns for the risk for pain and complications, the latter being said to be somewhat higher in small children. Several stated that when the indication for surgery is weak, then the risk of complications, however small, ought to weigh stronger. ‘Even though the risk is small, it is there’, one said. Furthermore, the procedure involved mutilation of a healthy body part. The informants pointed out that there were pros and cons that had to be weighed in the decision to use either general anaesthesia, which always involves a certain risk, or local anaesthesia, which would increase the risk of pain and thus potential psychological repercussions.

Concerning *justice* and fair priority setting, the informants pointed out that ritual circumcision uses finite capacity and resources. The procedure should be given a particularly low priority because ill patients, sometimes suffering from serious conditions, were waiting to be operated upon. Informants gave the impression that their departments were very busy and that they faced large workloads. This impression was corroborated by several department heads’ initial reluctance to allow their physicians to spend valuable time being interviewed for the study.

The urologists also discussed potential arguments *for* ritual circumcision being offered in their departments. Around half thought that the deep cultural and religious embeddedness and significance of ritual circumcision counted in favour of the procedure being legal, but still wanted it to be confined to the private sector. For these informants, respect for parental freedom of religion was an important factor. Other informants saw it differently; as one stated,

The fact that it is important for the group for religious reasons, I don’t see that as my problem as a doctor. If one had a religious group who demanded amputation of a foot, then one wouldn’t do it. One would never accept a corresponding intervention which did not have the same deep cultural roots.

Several thought that all things considered it would be better if the surgery was performed in a hospital, and not in an unsuited venue with unhygienic and otherwise less than adequate conditions. One stated, ‘the parents will get the surgery done anyway, and then it should be done by someone who is competent’. Pointing out that this line of reasoning – harm reduction – was a main justification for the government’s new policy, one informant thought this reference to the child’s interests to be paradoxical, as he claimed the procedure in itself would be contrary to the child’s rights. The informants were clear that the procedure should rather be done in the private sector than in public hospitals.

### Acting against one’s convictions or professional judgment

Several claimed that the state or the hospital cannot instruct surgeons to perform interventions that they do not deem medically indicated: ‘As a surgeon, it is a bit fundamental that no one can tell me that I should operate on someone’. It would be wrong to be ‘forced [to do] something one thinks is wrong’. Some thought that doctors have a right to refuse involvement in actions that are not medically indicated, and that the state should not dictate the contents of professional practice. As one said, ‘the very fundamental principle that one must treat the patient in accordance with what is best for the patient – that is such an unassailable thing about being a doctor. If it should be overridden by [the Minister of Health] – *that* does not work.’

The urologists were asked to reflect on an imaginary scenario where they in spite of their stance would be pressured to perform ritual circumcision. How would this impact them? Responses varied and some were initially reluctant to speculate. Some would see such pressure as abuse, betrayal, or as ‘extremely unpleasant’. Others used milder descriptions, such as feelings of powerlessness or irritation. Four informants invoked the concept of conscience, claiming that it would lead to, for instance, ‘a notch in my conscience’ or ‘some pangs of conscience’.

The informants were also asked to reflect on whether the dilemma of circumcision – and the scenario in which they were pressured to perform circumcision – concerned them as professionals only, or also as private individuals. Here also, reflections varied. Some stated that professional self-image was involved, but most added that it also touched on personal ethics. Some mainly directed the problem ‘outwards’, emphasizing that their main concern was not to harm the patient. Yet most also emphasized the injury to themselves of having to act against their conviction. One stated: ‘But if one were put to do something one thinks is wrong on principle, then it would change one as a person. Not just as a urologist, but as a human being … No matter what you do, you have to answer for why you do it.’ Another put it this way:

You can’t just say it’s the job or not the job, or [either] the job or [something] private. And that’s much of our profession also, we deal a lot with people, and a lot of things that one can think of at home and in private. It’s not possible to separate fully, then … So, this is somewhat important to me, then, that [our work] has a lot to say in private [life] as well. Because one is dealing with humans and health and life and death [in one’s work]. And those are big things.

Many of the informants considered whether they would have yielded to pressure; all stated that they would have been willing to perform circumcision if they had been threatened with loss of authorization as physicians, or with being fired without the prospect of another job.

### Refusing to perform circumcision as conscientious objection

Informants were invited to reflect on the relation of their own refusal to circumcise to another controversy which had received a lot of public attention in Norway recently, doctors’ conscientious objection to performing or referring for abortions. Some mentioned this parallel spontaneously. Overall, roughly half of informants mainly emphasized potential differences between the two cases, whereas the other half mainly emphasized similarities.

About half of the informants argued that stronger reasons to object were present in the case of a doctor who would be reluctant to perform abortion, than in their own case of reluctance to perform circumcision. Abortion was portrayed as a life-and-death issue, and ethical and religious opposition to abortion as more fundamental than their own opposition to circumcision: ‘I suppose it would feel heavier, if you’re opposed to it, to perform a late abortion in week 20 than to remove two grams of foreskin [laughs]’. Three other informants emphasized other differences: In abortion, the patient is competent and has requested the procedure, and failure to perform abortion can have large negative consequences for the woman’s life; whereas in circumcision, CO does not place the patient at risk for suffering – rather on the contrary. These differences pointed towards CO to abortion being *more* problematic than CO to circumcision, according to these informants.

A similarity or parallel pointed to by five informants was that in both cases, it is important for doctors not to be overridden by others, and that this is a fundamental principle for the physician profession. Furthermore, each professional has an individual responsibility for one’s own actions. One said,

I think it’s the same principle [in CO to abortion and circumcision, respectively]. People can have different consciences and think that the one is OK and the other is not OK. In ethical questions it is important that there is room to object to such things that are obviously ethically problematic for the business that you are doing. There, those things that have been discussed [i.e., abortion and circumcision] are genuine [i.e., important, relevant] issues. After all, we [also] do a lot of ungrateful work that is inconvenient. But there is no question of objecting to that.

### Collective support

The informants portrayed their relationship to department and hospital management and to colleagues as unproblematic. They had been met with understanding, and several worked in departments where department heads had been proactive in offering them the opportunity to abstain from performing circumcision. An atmosphere of understanding, practical adaptations and professional consensus in the ward had made it easier for the informants to take their stance and abide by it.

On the other hand, although informants were satisfied that the Norwegian Medical Association had taken a stance of opposition to circumcision, all but one thought that this support had not been of any significance for them in practice. The other informant stated that conscientious refusals are harder when you are on your own, but that the support of one’s professional organization makes it easier.

## Discussion

### Ethical reasoning about refusing to perform ritual circumcision

The opening questions in the interview guide asked informants to reflect on why they refused to participate in circumcision. Notably, the professional-ethical reflection of our informants zoomed in on the question of ritual circumcision itself – whether it is acceptable or not, and whether public hospitals should perform it. It was only after repeated prompting that they would consider the questions that lie on a higher level of abstraction: whether a physician who is opposed to circumcision ought to refuse to perform it, and whether colleagues, institutions and society ought to accept or tolerate such refusals. In a survey examining the Norwegian general population’s attitudes towards CO there were also signs of conflation of these issues [11, 12]; there, many respondents appeared to have answered the question of whether CO for procedure *x* ought to be tolerated mainly based on what they themselves thought of *x*. Plainly, however, even granted that *x* is morally problematic, it is a further question – and arguably a quite different one at that – whether refusals to perform *x* by a certain health professional in a certain health service ought to be tolerated [13–15].

### Professional and conscience-based refusals

In his influential discussion of conscientious objection, Mark R. Wicclair distinguishes rather sharply between conscience-based and professional refusals to provide healthcare services [16]. A conscience-based refusal (for instance, to perform abortion) springs from the professional’s *personal* ethical (or religious) norms, whereas the basis for a professional refusal (for instance, to perform a certain surgical procedure because it is too risky) is *professional* norms and standards. In the first case, the moral tenets involved are likely to be fundamental and belong to the professional’s core moral beliefs. In the second case, the moral tenets are professional norms that are not an integral part of the person’s moral identity in the same way. Because of this difference, being forced to act against one’s conviction is far more serious in the first case: the conscientious objector is likely to experience this as an act of self-betrayal, leading to emotions such as guilt, remorse and shame. The professional who is forced to transgress professional norms, on the other hand, performs an act that is (merely) morally and/or professionally inappropriate, and is likely to experience moral distress [16, 17].

Drawing on an example case, Magelssen challenged whether the distinction between conscience-based and professional refusals is really this sharp in practice [17]. If we take Wicclair’s theory to be a hypothesis, the present study lends itself well to testing it. Because the physicians as we have seen base their opposition to circumcision on professional norms, their refusal to perform circumcision is an instance of professional refusal to provide services.

The results conform well with Wicclair’s analysis in some respects, but not in others. When informants give reasons for opposing circumcision that map onto the four traditional principles of healthcare ethics, this shows that their basis for refusing is the breach of professional norms that circumcision appears to involve. Yet, as regards to the centrality of the moral norms for the informants, several gave the impression that the professional norms were also important for them personally and challenged the distinction between the professional and the personal. Half of the informants appeared to connect professional ethics principles and (personal) conscience. Several invoked the concepts of conscience and freedom of conscience in the course of the interviews. When asked how they would have experienced being forced to perform circumcisions, most gave descriptions that would fall under the category of moral distress (‘irritation’, ‘feeling of powerlessness’) [18], yet some also indicated more serious reactions and consequences that go beyond what is typically described as moral distress (e.g., ‘abuse’, ‘betrayal’).

If conscience is the faculty of judging the moral character of acts, then both professional and ‘personal’ moral norms can contribute to the content of conscience [17]. Both sets of norms can be sources of moral judgments. Throughout a professional’s career, perhaps some professional moral norms can assume such importance for him or her that they end up being ‘assimilated’ or ‘integrated’ into one’s core moral norms, becoming partly constitutive of one’s moral identity not only as a professional but as a person? One often hears physicians state that they have much of their identify in their practice of medicine (and that retirement might precipitate a crisis of identity). If their professional role indeed shapes who they are as persons, then perhaps it is apt to describe these physicians’ professional and personal (moral) identity as having become partly fused throughout the course of the career, so that these can no longer be clearly separated. One could then also speculate that when professional norms are sufficiently important (especially in life-death situations), then violating them can be just as problematic for the professionals as violating ‘personal’ moral norms. In this perspective, one explanation that most of the physicians in our study would experience ‘mere’ moral distress and not more severe reactions could be *not* that their stance is based on ‘mere’ professional norms, but that ritual circumcision is a somewhat less significant moral problem than life-and-death issues (as some of the informants also point out).

Another perspective here is that not just any professional norm is involved in this case. Beneficence, when specified as ‘act in the child’s best interest’, is of course a pervasive and central norm also outside of healthcare. The prospect of having to act in violation of this norm will be experienced as seriously morally challenging whether the context is within professional activity in healthcare, or in everyday life.

In sum, although Wicclair’s distinction between conscience-based and professional refusals can be upheld analytically, in practice the phenomena and their consequences for the professional might not necessarily differ.

### Comparison with CO to abortion

Conscientious objection to abortion is a ‘classic’ example of refusals based on ‘personal’ core moral norms. An interview study with Norwegian general practitioners (GPs) who refused to refer to abortion provides an instructive contrast with the present study [19]. For the GPs refusing referrals to abortion, their stance was especially justified by the inviolability of life and their associated Christian worldviews. The GPs experienced it as more or less impossible to have to act against their beliefs, but had nevertheless found compromises in how they organized their work. The way the informants discussed their moral viewpoints and the ethics of abortion lent credence to the emotional gravity the informants experienced.

Compared to this, our informants cannot be said to express an equally strong emotional commitment. Several of them show frustration and resentment about the new law on circumcision. For some, the issue has become personally serious. Yet, compared to the study on CO to abortion, in our case the moral gravity and felt seriousness appear to be significantly lower. This is also something many of the informants themselves emphasize: several of them point to the case of CO to abortion as a comparison, then proceed to say that the circumcision case does not impact them in the same degree as abortion impacts colleagues who are opposed to *it*. As they themselves portray it, ritual circumcision is a less significant intervention. Perhaps this is also a reason why most informants believe that circumcision after all should be legal.

### Ritual circumcision as a problem of professional ethics

The study shows how the urologists reason about ritual circumcision as a problem of professional ethics. Their arguments against the practice echo public debate; autonomy, harm, beneficence and justice are the common headings – harm appearing to be especially important. Yet, the informants’ professional background, role and practical know-how also enable them to deepen and exemplify the arguments in significant ways. Two examples mentioned above were, first, the dilemma in choosing between general or local anaesthesia, both of which had significant downsides. Second, practical priority setting in the workday, where the urologists’ view was that their workloads were large, waiting lists were long, and that prioritizing circumcision would make patients with genuine needs wait even further.

The ethical problem under scrutiny is an atypical CO case also in the way that the objectors here do not belong to a moral or religious minority; on the contrary, the informants have the support of a majority of the public (in a recent study, 80% of citizens polled supported CO to ritual circumcision [11]), in addition to many colleagues and department heads. None of the informants had had to ‘fight’ for their stance, and their departments had accommodated their refusals to partake. Perhaps this contributes to explaining why most of the informants would not readily call themselves ‘conscientious objectors’ or invoke concepts such as conscience or moral integrity; having experienced societal and collegial support, they have felt it sufficient to refer to the norms of the professions, and have not had any need of invoking frameworks of CO and minority rights, as the colleagues from Marquette, USA, apparently had [9].

### Strengths and limitations

The number of informants is small; we were unable to recruit any more. A self-selection bias where those more interested in the topic were more likely to respond, cannot be ruled out. Our impression is that potential informants were not reluctant to partake per se, but were unable or unwilling to prioritize participation in the study due to large workloads. As noted above, several department heads which we contacted did not want to forward the invitation to participate to their doctors because the doctors were pressed for time. The informants’ opposition to circumcision and their reasoning correspond well with how doctors’ views have been presented in the media [3]. The study only includes urologists, and not the other specialty which performs ritual circumcisions in Norway (paediatric surgeons). However, six hospitals in different geographical regions were represented. It would also have been relevant to study the viewpoints of nurses, who according to the law also have a right to refrain from participating in ritual circumcision.

## Conclusion

The study has explored urologists’ reasoning and justification for refusing to perform ritual circumcision. The physicians are unanimous in grounding their opposition to the procedure in professional standards and norms, based on fundamental tenets of professional ethics. While there is homogeneity in the group when it comes to this reasoning, there are significant variations as to how deeply the matter touches the urologists on a personal level. About half of them connect their stance to their personal integrity, and state that performing the procedure would go against their conscience and lead to pangs of conscience. It is argued that professional moral norms sometimes might become more or less ‘integrated’ in the professional’s core moral values and moral identity. If this is the case, then a distinction between conscience-based and professional refusals to certain healthcare services cannot be drawn as sharply as it has been.

## Supplementary information

**Additional file 1.** Interview guide.

## Data Availability

In order to protect informants’ anonymity, the data (transcripts) will not be shared.

## Abbreviation

CO: Conscientious objection
