# Supplementary material for: Refusals to perform ritual circumcision: a qualitative study of doctors’ professional and ethical reasoning
Source: BMC Med Ethics. 2020 Jan 10;21:5. doi: 10.1186/s12910-020-0444-0 (PMC6954583; doi:10.1186/s12910-020-0444-0)
Supplement: Supplementary file 1 — Additional file 1. Interview guide. [file 12910_2020_444_MOESM1_ESM.docx]

**“***Refusals to perform ritual circumcision: A qualitative study of doctors’ professional and ethical reasoning” (Litleskare et al.)*

**Interview guide**

1. What are your reasons for refusing to perform ritual circumcision?

- Why does this reasoning lead to an active refusal to perform, and not just a principled opposition?
- Have your views changed at some point; if so, why?
- Do you think that circumcision should not be offered in Norwegian hospitals at all, or do you accept it as long as you can opt out?

2. Tell me about a situation (for example, in clinical practice or in your employment relationship) where you experienced that your position was put to the test.

- What was the most personally challenging about the situation?

3. How would you have experienced being forced/pressed to perform circumcision anyway?

4. If the hospital’s ceased to tolerate refusals to perform circumcision, how consistent would you be in practice? What compromises would you consider accepting?

5. What counter-arguments to refusing do you see, and how do you weigh these?

6. What similarities and differences do you see with other conscientious objections in healthcare, such as refusals to perform an abortion?
